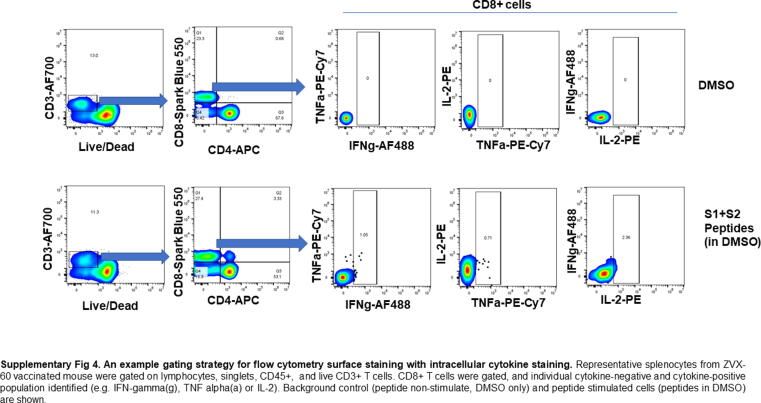# Secreted heat shock protein gp96-Ig and OX40L-Fc combination vaccine enhances SARS-CoV-2 Spike (S) protein-specific B and T cell immune responses

**DOI:** 10.1016/j.jvacx.2022.100202

**Published:** 2022-08-03

**Authors:** Laura Padula, Eva Fisher, Katelyn Rivas, Kristin Podack, Daniela Frasca, Jonah Kupritz, Matthew M. Seavey, Padmini Jayaraman, Eric Dixon, Rahul Jasuja, Natasa Strbo

**Affiliations:** a Department of Microbiology and Immunology, Miller School of Medicine, University of Miami, Miami, FL, USA; b Heat Biologics, Inc. Morrisville, NC, USA

**Keywords:** OX40L, Heat shock protein, Gp96, Vaccine, SARS-CoV-2 protein S, B cells, Antibody, TFH cells, CD8 T cells

## Abstract

Encouraging protection results from current mRNA-based SARS-CoV-2 vaccine platforms are primarily due to the induction of SARS- CoV-2- specific B cell antibody and CD4 + T cell. Even though, current mRNA vaccine platforms are adept in inducing SARS-CoV2-specific CD8 + T cell, much less is known about CD8 T cells contribution to the overall vaccine protection. Our allogeneic cellular vaccine, based on a secreted form of the heat-shock protein gp96-Ig, achieves high frequencies of polyclonal CD8 + T cell responses to tumor and infectious antigens through antigen cross-priming *in vivo*. We and others have shown that gp96-Ig, in addition to antigen-specific CD8 + T cell anti-tumor and anti-pathogen immunity, primes antibody responses as well. Here, we generated a cell-based vaccine that expresses SARS-Cov-2 Spike (S) protein and simultaneously secretes gp96-Ig and OX40L-Fc fusion proteins. We show that co-secretion of gp96-Ig-S peptide complexes and the OX40L-Fc costimulatory fusion protein in allogeneic cell lines results in enhanced activation of S protein-specific IgG antibody responses. These findings were further strengthened by the observation that this vaccine platform induces T follicular helper cells (TFH) and protein-S -specific CD8 + T cells. Thus, a cell-based gp96-Ig vaccine/OX40-L fusion protein regimen provides encouraging translational data that this vaccine platform induces pathogen-specific CD8+, CD4 + T and B cell responses, and may cohesively work as a booster for FDA-approved vaccines. Our vaccine platform can be rapidly engineered and customized based on other current and future pathogen sequences.

## Highlights

• gp96-Ig-S-OX40L-Fc vaccine enhances S-specific IgG responses.
• gp96-Ig-S-OX40L-Fc vaccine enhances TFH cell responses.
• gp96-Ig-S-OX40L-Fc vaccine enhances lungs S-specific CD8 + T cell responses.

## Graphical abstract

A cell-based vaccine co-secreting gp96-Ig, protein S1 and OX40L-Fc leads to activation of dendritic cells (DC) and cross-presentation of gp96-chaperoned protein S peptides to CD8+ T cells. Co-secretion of OX40L-Fc further augments CD8+T cell activity through OX40/OX40L axis. Secreted protein S1 is endocytosed by activated DC and S1 peptides are presented within the MHC class II molecules to TFH cells that also receive co-stimulatory signals through OX40L-Fc. In addition, B cells recognize secreted protein S1 through BCR and present S1 peptides within the MHC class II molecules to TFH cells. TFH cells through OX40/OX40L axis provide essential signals to promote B cell differentiation into memory B cells and long-lived plasma cells, and secretion of high-affinity specific antibodies. Local modulation of the gp96-Ig vaccine microenvironment by OX40L-Fc has the advantage to enhance immunological protection through providing additional support to CD8+ T cell and immunoglobulin antiviral response in the lungs and simplifying the clinical translation of such combination immunotherapies into humans.

**Figure:**
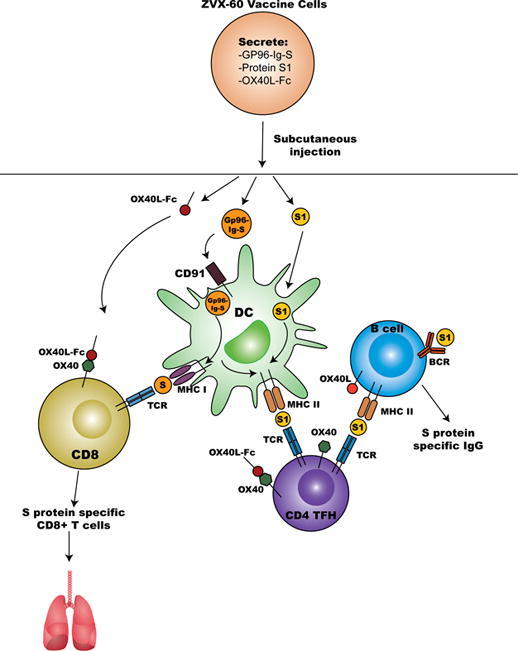

## Introduction

Over the last two decades, numerous animal and human studies have confirmed that the secreted heat shock protein gp96-Ig vaccine platform is effective in stimulating a robust cellular immune response against tumor and pathogen-derived antigens [1], [2], [3], [4], [5], [6], [7], [8], [9]. The endoplasmic reticulum heat shock protein, gp96 also known as Grp94, has unique intracellular chaperone properties that allow it to bind to a variety of endogenous peptides including tumor and pathogen-derived peptides [10], [11]. After these gp96-peptide complexes are released from damaged (infected) cells or administered via vaccination (gp96-Ig), they are internalized by antigen presenting cells (APCs) through the endocytic receptor CD91. This stimulates cross-presentation of chaperoned peptides as well as activation of nuclear factor (NF)-KB, release of pro-inflammatory cytokines and up-regulation of co-stimulatory molecules [5], [12], [13]. As a result, APCs activated by gp96 undergo maturation and become highly efficient in priming CD8 + and CD4 + T cell responses [12], [14], [15]. Recently, we confirmed that gp96-Ig, secreted from allogeneic cells expressing full-length SARS-CoV-2 Spike (S) protein, generates powerful, polyepitope S protein-specific CD4 + and CD8 + T cell responses in both lung interstitium and airways [1]. The emergence of SARS-CoV-2 variants has highlighted the need for T-cell immunity against this deadly virus. CD4 + T-helper responses are required for effective B cell help in generating neutralizing antibodies, and CD8 + T-cells are required to clear virus-infected cells, particularly in the early stage of SARS-CoV2 infection [16] as well as in the early protection window after prime vaccination [17]. We generated a cell-based vaccine expressing gp96-Ig, SARS-CoV-2 S protein, and OX40L-Fc, called ZVX-60, to induce potent memory T-cell responses and aid in antibody production.

T follicular helper (TFH) cells are a subset of CD4 + T cells residing in secondary lymphoid organs. They are defined by their expression of the transcription factor B cell lymphoma 6 (Bcl6) and several cell surface markers including CXCR5, PD1, and ICOS [18], [19]. They play a critical role in protective immunity by providing continuous co-stimulatory help to B cells and driving high-affinity antibody production against pathogens, but they have also been implicated in pathogenesis of some autoimmune diseases [20]. Recently, it was reported that elevated frequencies of activated TFH cells were found in the blood of patients with non-severe COVID-19 symptoms [21], [22]. Furthermore, high frequencies of activated circulatory TFH (cTFH) cells correlated with lesser disease severity in COVID-19 patients [23]. Given the importance of humoral immunity in fighting SARS-CoV-2 and the importance of TFH cells in stimulating germinal center (GC) B cell responses and high-affinity antibody production [22], [24], [25], [26], [27], there is high demand for development of improved COVID19 vaccine strategies that target TFH cells.

The OX40 ligand (OX40L, CD252, TNFSF4), belonging to the TNF superfamily, is induced on professional APCs [28], [29], [30], and binds to OX40 (CD134, TNFRSF4) on activated CD4 + T cells, CD8 + T cells, NK cells and NKT cells [22], [31], [32]. The OX40/OX40L axis plays a major role in regulating CD4 + and CD8 + T cell clonal expansion, memory development and maintenance [31], [33], [34], [35], [36]. Furthermore, OX40 blocks natural T regulatory (Treg) cell activity and antagonizes generation of inducible Tregs [37]. It has also been confirmed that the OX40L/OX40 axis promotes the differentiation of naive and memory T cells into TFH while blocking the suppressive function of Tregs and regulatory TFH cells [38]. Since one of the most important functions of TFH cells is to provide help to B cells, providing OX40L during antigen priming supports this function by promoting TFH cell differentiation and cell survival resulting in the potent B cell responses and antibody affinity maturation. Here, we show that a vaccine that co-expresses SARS-CoV-2 protein S, OX40L-Fc fusion protein and secreted gp96-Ig vaccine, results in enhanced activation of S protein-specific IgG antibody responses, T follicular helper cells (TFH) and protein-S -specific CD8 + T cells.

## Methods

### Generation of vaccine cell lines

Human lung adenocarcinoma cell lines (AD100)[39], [40] (source: University of Miami, FL, USA) were transfected with 2 plasmids: B45 encoding gp96-Ig (source: University of Miami) and pcDNA™ 3.1(-) (Invitrogen), encoding full-length SARS-CoV-2 Spike (S) protein gene (Genomic Sequence: NC_045512.2; NCBI Reference Sequence: YP_009724390.1 GenBank Reference Sequence: QHD43416) as previously described [1]. In this manuscript, Spike (S) protein is referred to as “S protein”. The OX40L open reading frame was synthesized by Gibson assembly and inserted 3’ to IgG4-Fc. OX40L-IgG4-Fc cDNA was cloned into the pCEP4 expression cassette. cDNA has KpnI restriction site flanking the Kozak sequence and 3’ of the non-sense codon has XhoI restriction site. AD100 cells were simultaneously transfected with B45 and pcDNA 3.1 plasmids by lipofectamine (Invitrogen) following the manufacturers’ protocol. Transfected cells were selected with 1 mg/mL of G418 (Life Technologies, Inc.) and 7.5 mM of L-Histidinol (Sigma Chemical Co., St. Louis, MO, USA). After the stable transfection, a cell line was established, and the third plasmid, pCEP4 encoding OX40L-Fc, was introduced using the same transfection method as above. For selection, 200 μg/ml of Hygromycin B Gold (InvivoGen, Cat#ant-hg) was added to the above selection cocktail. Single cell cloning by limiting dilution assay was performed, and all cell clones were first screened for gp96-Ig production and then for OX40L-Fc production and protein S expression. The cell clone ZVX60 had the highest expression of gp96-Ig, S protein, and OX40L-Fc.

### Western blotting and Enzyme-Linked immunosorbent assay (ELISA)

SARS-CoV-2 S protein expression was verified by SDS-page and Western blotting using mouse monoclonal IgG1 kappa anti S1 protein, clone CR3022 (MBS434277) (MyBiosource) at 1/500 dilution and secondary antibody: HRP conjugated Anti Mouse IgG, Fc gamma, subclass 1 specific (Cat#115–035-205) (Jackson ImmunoResearch) at 1/5,000 dilution. S protein was visualized by an enhanced chemiluminescence detection system (Cat#34096) (SuperSignal West Femto, Thermo Scientific). Recombinant human coronavirus SARS-CoV-2 spike glycoprotein S1 (Fc Chimera) (ab272105) (Abcam) was used as a positive control (loaded 2.4 ug/lane).

One million cells were plated in 1 ml of growth medium for 24 h and secreted gp96-Ig production was determined by ELISA using sheep anti-human gp96 antibody (HSP90B1-R&D Systems, Cat. No. AF7606) as coating antibody, peroxidase conjugated goat anti-human IgG antibody as a secondary antibody (Abcam Ab7499), and purified gp96-Ig IgG1 as a standard. OX40L-Fc production was measured using hOX40-His (TNFRS4/CD134) (Acro Biosystems, Cat. No. OX0-H5224) as coating antibody and human OX40L Fc (TNFSF4) (Thermo Fisher Scientific (Invitrogen) Lot# 9090790) as a standard. A Peroxidase-conjugated Goat Anti-human IgG, Fc Gamma Fragment Specific antibody (Jackson ImmunoResearch Cat. No.: 109–035-098) was used as detection antibody. For Spike protein detection in the million cells supernatant, we used a Kit from Abclonal®, SARS-CoV-2 S1 + S2 ECD Protein ELISA kit. Cat. No.: RK04159 following the manufacturers’ protocol.

### Immunofluorescence (IF)

AD100-gp96-Ig-S cells were cytocentrifuged at 1000xg for 2 min onto charged microscope slides (1x10^5^ live cells per condition). Cytospins were fixed in pure cold methanol (VWR, Cat. No.: VW5868) for 5 min followed by 3x5 min washes with phosphate-buffered saline (PBS). The slides were left in blocking media (5% goat serum, 1% bovine serum albumin (BSA), 0.1% Tween in PBS) at room temperature for 2 h. All antibodies and controls were diluted in blocking media. The primary antibody, rabbit monoclonal IgG anti-SARS-CoV-2 spike S1 antibody (HL6) from GeneTex (GTX635654) and rabbit isotype control (Abcam Ab172730) were added in 1/1000 dilutions (1ug/ml) and incubated overnight at 4 °C in a dark moisture chamber. The next day, slides were washed 3 times for 5 min with PBS and incubated for 90 min at room temperature with goat anti-rabbit IgG Alexa Fluor 594 (Invitrogen cat # A11012), secondary antibody diluted 1/1000 (2ug/ml). The slides were rinsed 3 times and mounted with Prolong Gold antifade reagent with DAPI (Invitrogen; Catalog #36935), mounted with a coverslip and allowed to cure for 24 h before acquiring on a KEYENCE microscope (BZ-X Viewer). The following filter cubes were used: DAPI (for nuclear stain) and Texas Red (for protein S1).

### Animals and vaccination

Mice used in this study were colony-bred mice (C57Bl/6) and human leukocyte antigen (HLA)-A02-01 transgenic mice (C57BL/6-Mcph1Tg (HLA-A2.1)1Enge/J, Stock No: 003475) purchased from JAX Mice (Jackson Laboratory for Genomic Medicine, JAX, Farmington, CT, USA). Homozygous mice carrying the Tg (HLA-A2.1)1Enge transgene express human class I major histocompatibility complex (MHC) Ag HLA-A2.1 (JAX mice Stock No.: 003475/ HLA-A2). [41]. The animals were housed and handled in accordance with the standards of the Association for the Assessment and Accreditation of Laboratory Animal Care International under University of Miami Institutional Animal Care & Use Committee-approved protocol. Both female and male mice were used at 6–10 weeks of age.

Equivalent numbers of AD100-gp96-Ig-protein S and AD100-gp96-Ig-S-OX40L-Fc cells that produce 0.5, 1 or 2 μg/ml gp96-Ig or PBS were injected via the subcutaneous (s.c.) route in C57Bl/6 and HLA-A2 transgenic mice. Mice were vaccinated on day 0 and 14 and were sacrificed 5 days after vaccination (day 19). Spleen, lungs, and bronchoalveolar lavage (BAL) were collected and processed into single-cell suspensions.

### Spleen, bronchoalveolar lavage (BAL) cell collection and lung tissue cell isolation

For mouse samples, spleens were collected, and tissues processed into single-cell suspension. Leukocytes were isolated from spleen by mechanical dissociation and red blood cells were lysed by lysing solution. BAL was harvested directly from euthanized mice via insertion of a 22-gauge catheter into an incision into the trachea. Hanks’ Balanced Salt Solution (HBSS) was injected into the trachea and aspirated 4 times. Recovered lavage fluid was collected and BAL cells were gathered after centrifugation.

To isolate intraparenchymal lung lymphoid cells, the lungs were flushed by 5 ml of prechilled HBSS into the right ventricle. When the color of the lungs changed to white, the lungs were excised avoiding the peritracheal lymph nodes. Lungs were then removed, washed in HBSS, cut into 300-mm pieces, and incubated in Iscove’s Modified Dulbecco’s Medium containing 1 mg/mL collagenase IV (Sigma) for 30 min at 37° C on a rotary agitator (approximately 60 rpm). Any remaining intact tissue was disrupted by passage through a 21-gauge needle. Tissue fragments and the majority of the dead cells were removed by a 250-mm mesh screen, and cells were collected after centrifugation.

### Flow cytometry

Spleen from immunized and control animals were analyzed for B cell and TFH responses. 1–1.5 × 10^6^ cells were first labeled with the LIVE/DEAD Fixable Violet Dead Cell Stain Kit (Thermo Fisher Scientific, Waltham, MA, USA) and then resuspended in BD Fc Block (clone 2.4G2) for 5 min at room temperature prior to staining with a surface-stain cocktail containing the following antibodies purchased from BioLegend® (San Diego, CA, USA): APC/ Cy7 CD45, Clone: 30-F11; Alexa Fluor 700 CD3, Clone:17A2; APC CD4, Clone: RM4-5; Brilliant Violet 510 CD19, Clone: 6D5; PE/Dazzle 594 IgM, Clone: RMM-1; FITC CD21, Clone: 7E9; PE CD23, B3B4; PerCP-Cy5.5 CD43, Clone: 1B11, and PerCP-Cy5.5 CD93, Clone: AA4.1 (Supplementary Fig. 2).

### HLA-A02-01 pentamer staining

A total of 1-2x10^6^ spleen, BAL, or lung cells were labelled with the peptide-MHC class I pentamer-APC (ProImmune, Oxford, UK) and incubated for 15 min at 37° C. Cells were labelled with the LIVE/DEAD™ Fixable Violet – Dead Cell Stain Kit (Invitrogen, Carlsbad, CA, USA) and then stained with the following antibody cocktail: APCCy7 CD45, Clone: 30-F11; AF700 CD, Clone: 17A2; PECy7 CD4, Clone: RM4-5; FITC or Spark Blue 550 CD8, Clone:53–6.7; PE Dazzle CD69, Clone: H1.2F3; BV 605 CD44, Clone: IM7; BV510 CD62L, Clone: MEL-14, and PerCP/Cy5.5 CXCR6, Clone: K041E5. Spleen and lung cells that were stimulated overnight with peptide pools (as described under ex-vivo stimulation and intracellular staining) were fixed and permeabilized with Cytofix/Perm solution (BD) and then stained for intracellular cytokines: IFNγ, and IL-2. Cells were acquired on the SP6800 Spectral Cell Analyzer (Sony) and the data was analyzed using FlowJo software version 10.8. Data were analyzed using forward side-scatter single-cell gate followed by CD45, CD3, and CD8 gating, then pentamer gating within CD8 + T cells (Supplementary Fig. 3). These cells were then analyzed for expression of markers using unstained and overall CD8 + population to determine the placement of the gate. Single-color samples were run for compensation and fluorescence minus 1 control sample were also applied to determine positive and negative populations, as well as channel spillover.

### Peptide stimulation and intracellular cytokine staining

Spleen and lung lymphocytes from vaccinated and control animals were analyzed for protein S-specific CD8 + T cell responses 5 days after vaccination. One million cells were incubated for 20 h with 2 protein S peptide pools (S1 and S2, homologous to vaccine insert, UniProt: P0DTC2) (JPT Peptide Technologies, Berlin, Germany; PM-WCPV-S1). Peptide pools were combined (S1 + S2) and used at a final concentration of 1.25 ug/mL of each peptide, followed by addition of Brefeldin A (BD GolgiPlug™; BD Biosciences, San Diego, CA, USA) (10 ug/mL) for the last 5 h of the incubation. Stimulation without peptides served as background control (Supplementary Fig. 4). The results were calculated as the total number of cytokine-positive cells with background subtracted. Intracellular cytokine staining was performed as previously described [1]. Briefly, surface and intracellular antibodies were purchased from BioLegend® (San Diego, CA, USA): (APC)Cy7 CD45: Clone: 30-F11; AF700 CD3: Clone: 17A2; APC CD4: Clone:RM4-5; Spark Blue 550 CD8: Clone:53–6.7; PE Dazzle CD69: Clone:H1.2F3; Alexa Fluor 488 interferon (IFN) gamma: Clone: XMG1.2; PE interleukin 2 (IL-2): Clone: JES6-5H4; PE Cy7 tumor necrosis factor alpha (TNFα): Clone: MPG-XT22. BD. Cytofix/Perm fixation/permeabilization solution kit was used according to manufacturer instructions. Data were collected on Spectral analyzer SONY SP6800 instrument (Sony Biotechnologies, Inc, San Jose, CA, USA). Analysis was performed using FlowJo™ software version 10.8 (Tree Star Inc, Ashland, OR, USA). Cells were first gated on live cells, CD3 + T cells and antigen-responding CD8 + T cells (IFNγ, or IL-2, or TNFα-producing/expressing cells) were determined on the total CD8 + T cell population (Supplementary Fig. 4).

### Statistical analyses

All experiments were conducted independently at least 3 times on different days with n = 3–5 mice/group. Comparisons of flow cytometry cell frequencies were measured by the 2-way analysis of variance (ANOVA) test with Holm-Sidak multiple-comparison test, *p < 0.05, **p < 0.01, and ***p < 0.001, or unpaired T-tests (2-tailed) to compare the control group with each of the experimental groups (alpha level of 0.05) using the Prism software (GraphPad Software, San Diego, CA, USA). Welch’s correction was applied with the unpaired T test, when the p-value of the F test to compare variances was ≤ 0.05. Data approximately conformed to Shapiro-Wilk test and Kolmogorov-Smirnov tests for normality at 0.05 alpha level. Data were presented as mean ± standard deviation in the text and in the figures. All statistical analysis was conducted using GraphPad Prism 8 software**.**

## Results

### ZVX-60 vaccine cells express gp96-Ig, SARS-CoV2 protein S and OX40L-Fc

**Fig. 1:**
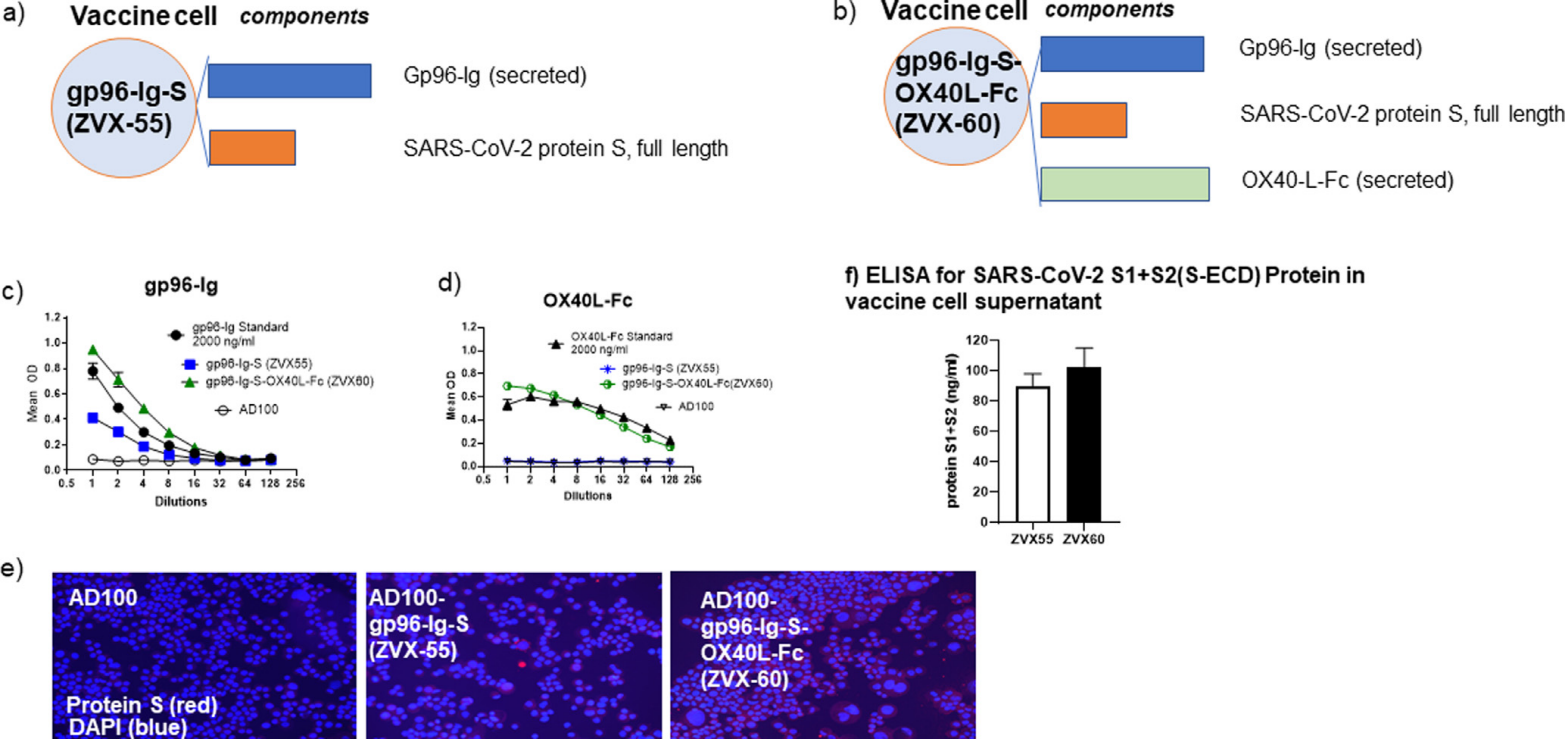
**Characteristics of the cell line expressing gp96-Ig, SARS-CoV-2 Spike (S) protein and OX40L-Fc.** Cell line (AD100) was transfected with plasmids encoding a) gp96-Ig and full length protein S and b) gp96-Ig, full length protein S and OX40L-Fc. c) Secreted gp96-Ig was measured in the cell supernatant by ELISA. One million cells were plated in 1 ml for 24 h. Purified gp96-Ig was used as standard d) Secreted OX40L-Fc was measured in the cell supernatant by ELISA. One million cells were plated in 1 ml for 24 h. Purified OX40L-Fc was used as standard e) SARS-CoV2 protein S expression was analyzed by immunofluorescence f) SARS-CoV2 protein S expression in supernatant was measured by ELISA. One million cells were plated in 1 ml for 48 h and purified SARS-CoV2 protein S was used as standard.

Cell-based secreted heat shock protein technology has been validated previously in animal and human studies [3], [6], [7], [42] and was recently employed as a SARS-CoV2 vaccine [1]. Using the strategy previously described and published [1] and summarized in Fig. 1**a**, we generated a cell-based secreted gp96-Ig vaccine (gp96-Ig-S) for COVID-19 by co-transfecting AD100 cells with plasmids encoding gp96-Ig and full-length protein S and selecting transfected cells with G418 and L-histidinol. Prior studies demonstrated potential synergy between various T cell costimulatory ligands and gp96-Ig based vaccines [36]. The human OX40L (CD252), the ligand for human OX40 (tumor necrosis factor receptor family member, TNFRSF4 or CD134), was re-engineered to express an OX40L-Fc fusion protein by 3′ insertion of IgG4-Fc in the open reading frame of OX40L [36]. We generated OX40L-Fc expressing vaccine cells, by triple transfection of AD100 with plasmids encoding gp96-Ig, SARS-CoV2 full-length protein S and fusion protein OX40L (Fig. 1**b**). We confirmed by ELISA that both stable transfected cell lines, gp96-Ig-S (ZVX-55) and gp96-Ig-S-OX40L (ZVX-60), secrete gp96-Ig into culture supernatants at a rate of approximately 1100 ng/mL/24 h/10^6^ ZVX-55 vaccine cells and 2200 ng/ml/24 h/10^6^ ZVX-60 vaccine cells (Fig. 1**c**). We also analyzed the production/secretion of OX40L-Fc from both vaccine cells (Fig. 1**d**) and we found that ZVX-60 secretes 2000 ng/mL/24 h/10^6^ cells. The non-transfected cell line, AD100 as well as ZVX-55 did not produce OX40L-Fc (Fig. 1**d**).

**Supplementary Fig. 1:**
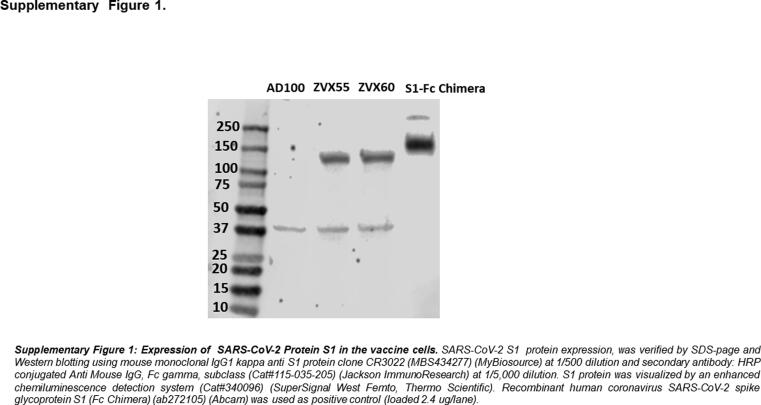


**Supplementary Fig. 2:**
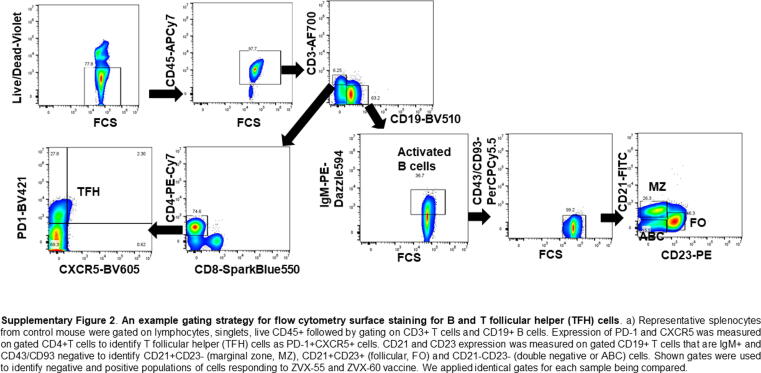


**Supplementary Fig. 3:**
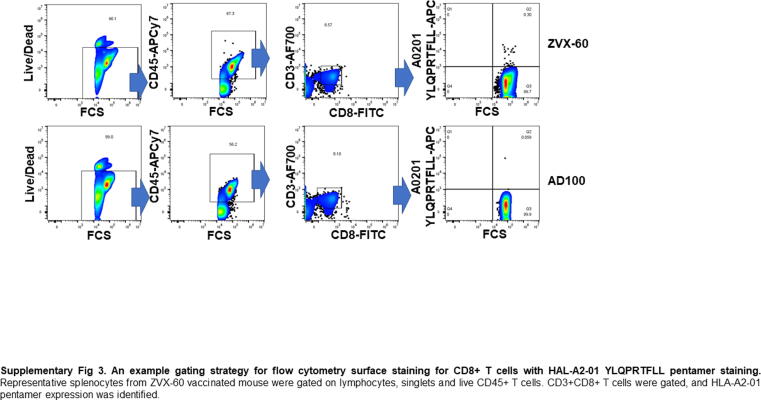


SARS-CoV2 protein S expression by the vaccine cells was confirmed by immunofluorescence (Fig. 1**e**), ELISA (Fig. 1**f**) and Western blotting (Supplementary Fig. 1). We observed cytoplasmic and transmembrane expression of full-length protein S only in AD100 transfected cell lines (ZVX-55 and ZVX-60) but not in the non-transfected AD100 cell line (Fig. 1**e**). Like the S proteins of other coronaviruses, the S protein of SARS-CoV-2 is cleaved into S1 and S2 proteins by cellular proteases, and the serine protease TMPRSS2 [43], [44]. We detected the soluble S1 protein by ELISA in the vaccine cell supernatant (Fig. 1**f**) and the amount of S1 protein in the supernatant of ZVX-55 and ZVX-60 was comparable (Fig. 1**f**).

We therefore confirmed the expression of all three protein components (gp96-Ig, OX40L-Fc and S protein) in transfected AD100 and used this cell line, termed ZVX-60, for immunogenicity studies as described below.

### Secreted gp96-Ig-S-OX40L-Fc (ZVX-60) vaccine increases S protein specific IgG and B cell responses

**Fig. 2:**
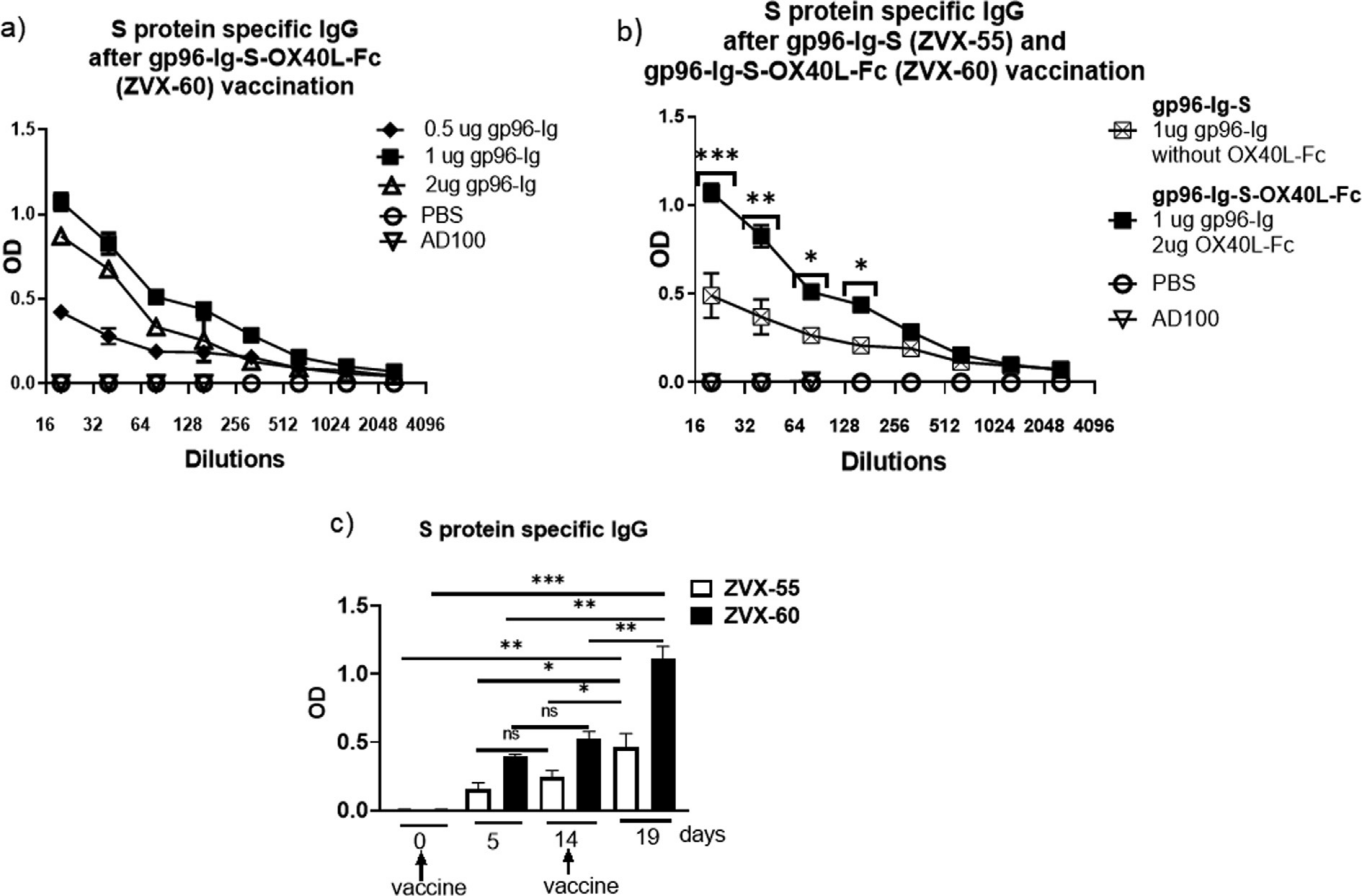
**Gp96-Ig and OX40L-Fc increase S protein specific IgG responses *in vivo*.** a) C57Bl6 mice were vaccinated at day 0 and 14 with different concentrations of cell-based gp96-Ig vaccine that expressed SARS-CoV-2 glycoprotein S and OX40L-Fc or with AD100 or PBS (controls). b) Mice were vaccinated at day 0 and 14 with 1 μg/ml of ZVX-55 and ZVX-60 or with AD100 and PBS (controls). Serum was collected 5 days after last vaccination, and S protein specific IgG response was analyzed by ELISA. c) Mice were vaccinated at day 0 and 14 with 1 μg/ml ZVX-55 or ZVX-60 and S protein specific IgG response in serum was analyzed at day 5, 14 and 19. Data represent 3 independent biological replicates per group and mean ± standard error. To compare control (ZVX55) with experimental (ZVX60) group (alpha level of 0.05) unpaired *t*-test (two-tailed) was applied, *p < 0.05, **p < 0.01, and ***p < 0.001.

We found that, when secreted, antigenic proteins expressed in vaccine cells together with gp96-Ig, can serve as a source of exogenous protein for B cell activation [6] and antibody production. To test the effect of different doses of ZVX-55 and ZVX-60 vaccines, we standardized the vaccination dose to a set amount of gp96-Ig secreted by 10^6^ vaccine cells within 24 h. In our dose–response experiments, we immunized mice with 0.5, 1 or 2 μg/ml of the ZVX-55 and ZVX-60 vaccine on day 0 and day 14 (Fig. 2). S protein specific IgG antibodies were measured in the serum samples of vaccinated animals, 5 days after the last dose (day 19). We found that animals vaccinated with 1 μg/ml gp96-Ig-S have the highest titers of S protein-specific IgG antibodies (Fig. 2**a**), whereas the mice vaccinated with the 2 μg/ml dose had slightly lower titers (Fig. 2**a**). When we compared the effect of the same dose of ZVX-55 to the ZVX-60 vaccine, we observed significant enhancement of S protein-specific IgG responses in ZVX-60 vaccinated mice (Fig. 2**b**). Furthermore, analysis of time course of S protein specific IgG responses showed that there is no statistically significant difference in the level of antibody responses after first dose of ZVX-55 and ZVX-60 vaccination on day 5 and day 14 post vaccination. However, 5 days after the second dose of the vaccine, we observed a significant increase in the level of antibodies, for both ZVX-55 and ZVX-60 (Fig. 2**c**).

**Fig. 3:**
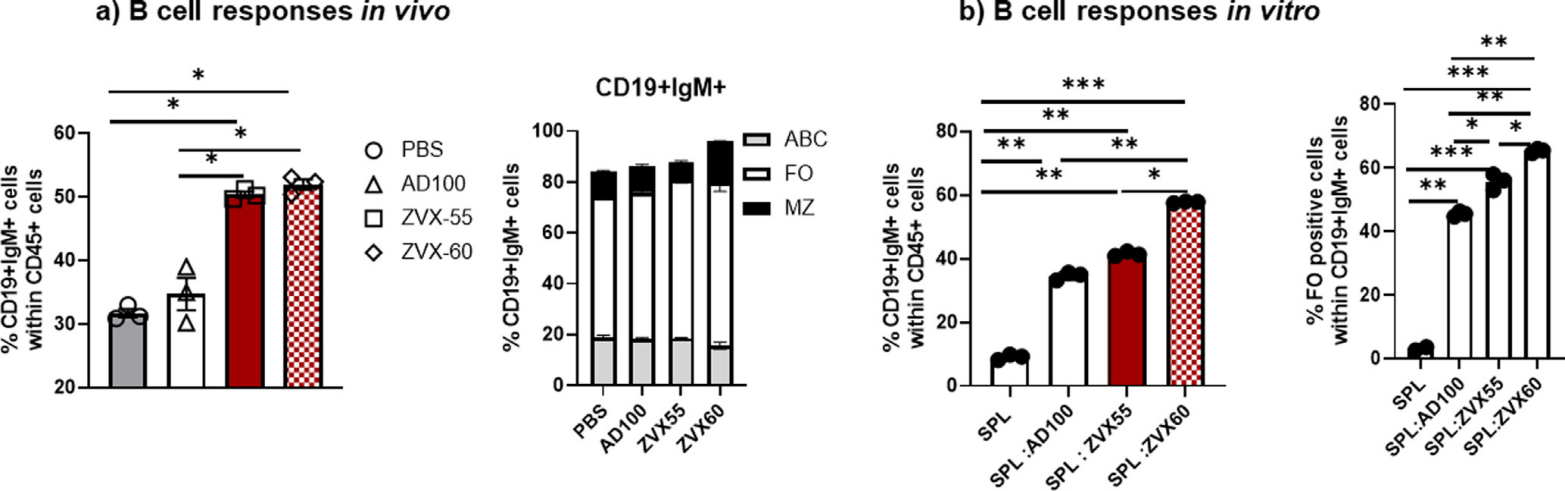
**Gp96-Ig and OX40L-Fc induce B cells responses.** C57Bl6 mice were vaccinated at day 0 and 14 with a cell-based gp96-Ig vaccine that expressed SARS-CoV-2 glycoprotein S (ZVX-55, 1ug gp96-Ig) and OX40L-Fc (ZVX-60,1 ug gp96-Ig) or with AD100 or PBS (controls). a) Spleen cells (SPL) were isolated from vaccinated and control mice 5 days after last vaccination, stained for CD45, CD3, CD19, IgM, CD21, CD23, CD49, CD93. Frequency of CD19 + IgM+ (activated B cells) and CD21 + CD23- (marginal zone, MZ), CD21 + CD23+ (follicular, FO) and CD21-CD23- (double negative or ABC cells) CD19 + IgM + cells was determined by flow cytometry. b) SPL were isolated from unvaccinated mice and co-cultured with vaccine cells (ZVX55 or ZVX60) and control cells AD100 at 5:1 ratio for 96 h. Frequency of activated B cells (CD19 + IgM + ) within total CD45 + T cells and frequency of FO (CD21 + CD23 + ) within CD19 + IgM + cells was determined by flow cytometry. Data represent 3 independent biological replicates per group and mean ± standard error. To compare > 2 experimental groups, 2-way analysis of variance (ANOVA) test with Holm-Sidak multiple-comparison test were applied, *p < 0.05, **p < 0.01, ***p < 0.001.

Since gp96-Ig-S induced S protein-specific IgG responses, we wanted to analyze the phenotype of B cell responses after vaccination. We found an increased frequency of activated B cells (CD19 + IgM + ) in ZVX-55 and ZVX-60 vaccinated mice compared to control mice (Fig. 3**a**), withboth vaccines inducing similar frequencies of activated B cells. Among activated B cells, we did not observe any statistically significant differences in the frequency of follicular (FO), marginal zone (MZ), or double negative B cells (also known as age-associated B cells, ABC) in mice vaccinated with ZVX-55 and ZVX-60 compared to the controls, PBS and AD100. (Fig. 3**a**).

To determine the effect of OX40L on B cell differentiation *in vitro*, we co-cultured spleen cells from non-vaccinated mice with the ZVX-55 and ZVX60 vaccine cells at ratio 5:1 for 96 h. We found that ZVX-55 induced similar frequencies of activated B cells (CD19 + IgM + ) when compared to the control, AD100 cells (not transfected with gp96-Ig-S) (Fig. 3**b**). Conversely, ZVX-60 induced a significant increase in the frequency of CD19 + IgM + cells as compared to ZVX-55 and the media control (SPL) (Fig. 3**b**). Furthermore, we confirmed that the predominant population of B cells in the co-culture with ZVX-55 or ZVX-60 vaccine cells are FO B cells (Fig. 3**b**).

Overall, vaccination with gp96-Ig-S-OX40L-Fc induced higher levels of S protein-specific IgG antibodies in the serum of vaccinated animals compared to gp96-Ig-S. Our vaccine can therefore successfully elicit a SARS-CoV2 protein S-specific antibody response, which could play a pivotal role in conferring robust immunity against SARS-CoV-2 infection.

### Secreted gp96-Ig-S-OX40L-Fc (ZVX60) vaccine enhances TFH cell responses

**Fig. 4:**
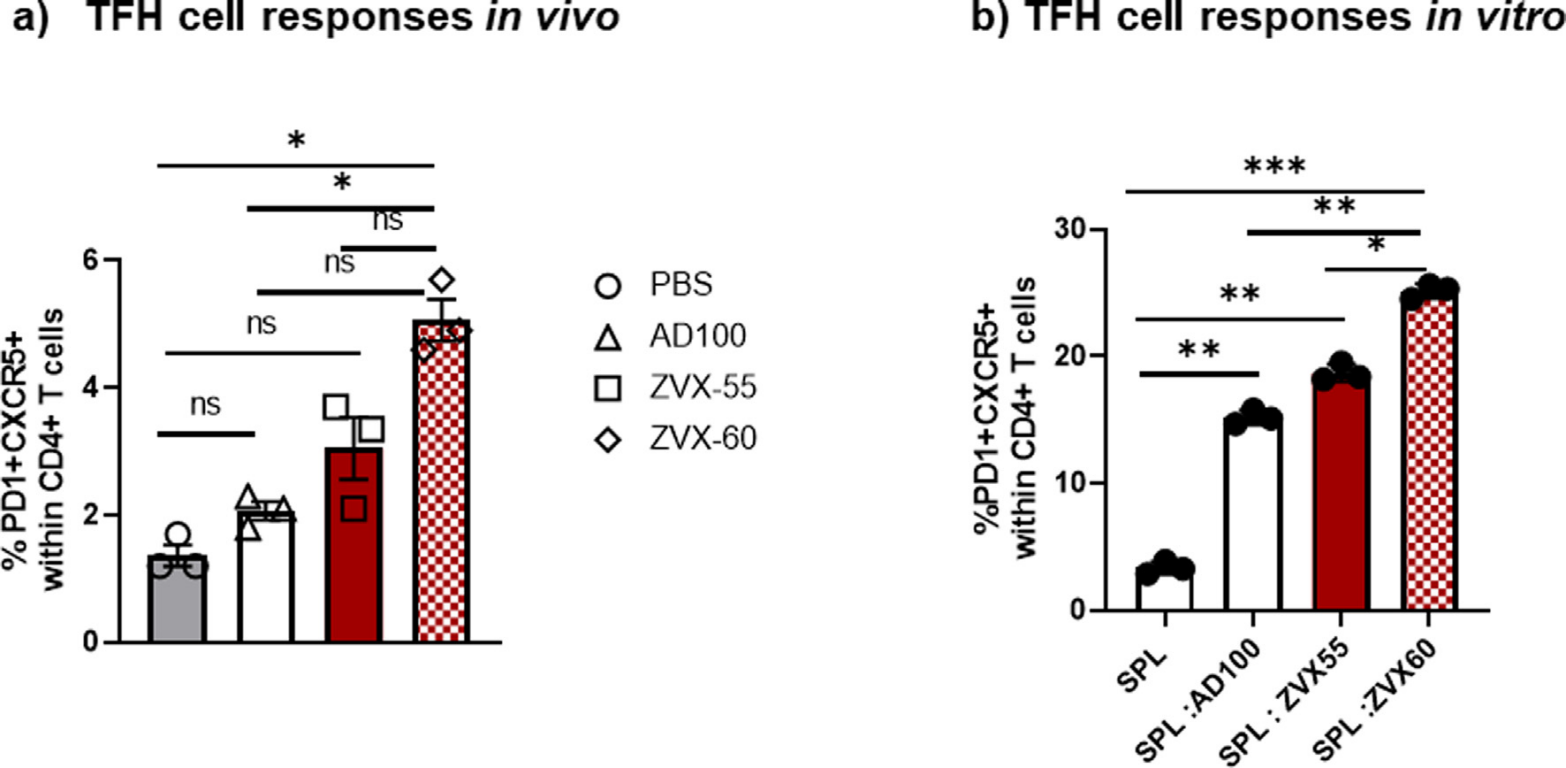
**Gp96-Ig and OX40L-Fc induce T follicular helper (TFH) cell responses.** C57Bl6 mice were vaccinated at day 0 and 14 with a cell-based gp96-Ig vaccine that expressed SARS-CoV-2 glycoprotein S (ZVX-55, 1ug gp96-Ig) and OX40L-Fc (ZVX-60,1 ug gp96-Ig) or with AD100 or PBS (controls). a) Spleen cells (SPL) were isolated from vaccinated and control mice 5 days after last vaccination, stained for CD45, CD3, CD4, PD1 and CXCR5. Frequency of PD1 + CXCR5+ (TFH cells) within CD4 + T cells was determined by flow cytometry. b) SPL were isolated from unvaccinated mice and co-cultured with vaccine cells (ZVX55 or ZVX60) and control cells AD100 at 5:1 ratio for 96 h. Frequency of TFH cells (PD1 + CXCR5 + ) within total CD4 + T cells was determined by flow cytometry. Data represent 3 independent biological replicates per group and mean ± standard error. To compare > 2 experimental groups, 2-way analysis of variance (ANOVA) test with Holm-Sidak multiple-comparison test were applied, *p < 0.05, **p < 0.01, ***p < 0.001.

TFH cells provide cognate help to B cells in establishing long-lived high affinity antibody responses [24], [26]. We analyzed TFH cell responses after ZVX-55 and ZVX-60 vaccination *in vivo* (Fig. 4**a**) and found that only ZVX-60 significantly increased the frequency of TFH cells in the spleen of vaccinated mice compared to non-vaccinated controls (Fig. 4**a**). We also observed higher frequencies of TFH cells in ZVX-55 vaccinated animals compared to non-vaccinated animals, however the difference in the vaccine-induced TFH frequencies was not statistically significant (Fig. 4**a**).

To test whether the vaccine can induce generation of TFH cells from CD4 + T cells, we set up a co-culture system with non-vaccinated SPL and vaccine cells (ZVX-55 and ZVX-60). Co-culture of SPL cells with un-transfected AD100 cells increased the frequency of TFH cells after 96hrs, compared to the SPL cells that were cultured in medium only (Fig. 4**b**). Co-culture of SPL with ZVX-55 vaccine cells did not further increase the frequency of TFH cells (Fig. 4**b**) while co-culture with ZVX-60 resulted in a significant increase in TFH cells compared to both SPL co-culture with AD100 and SPL co-culture with ZVX-55 (Fig. 4**b**).

Overall, ZVX-60 is reliable and represents a versatile vaccine platform for the generation of TFH cells. Our study confirms that the gp96-Ig-OX40L vaccine platform is a unique activator of cognate B cells through activation of TFH cells.

### Induction of SARS-CoV-2 protein S epitope-specific CD8 + T cells in the lungs and airways of gp96-Ig-S-OX40L-Fc (ZVX-60) vaccinated HLA-A2-transgenic mice

**Fig. 5:**
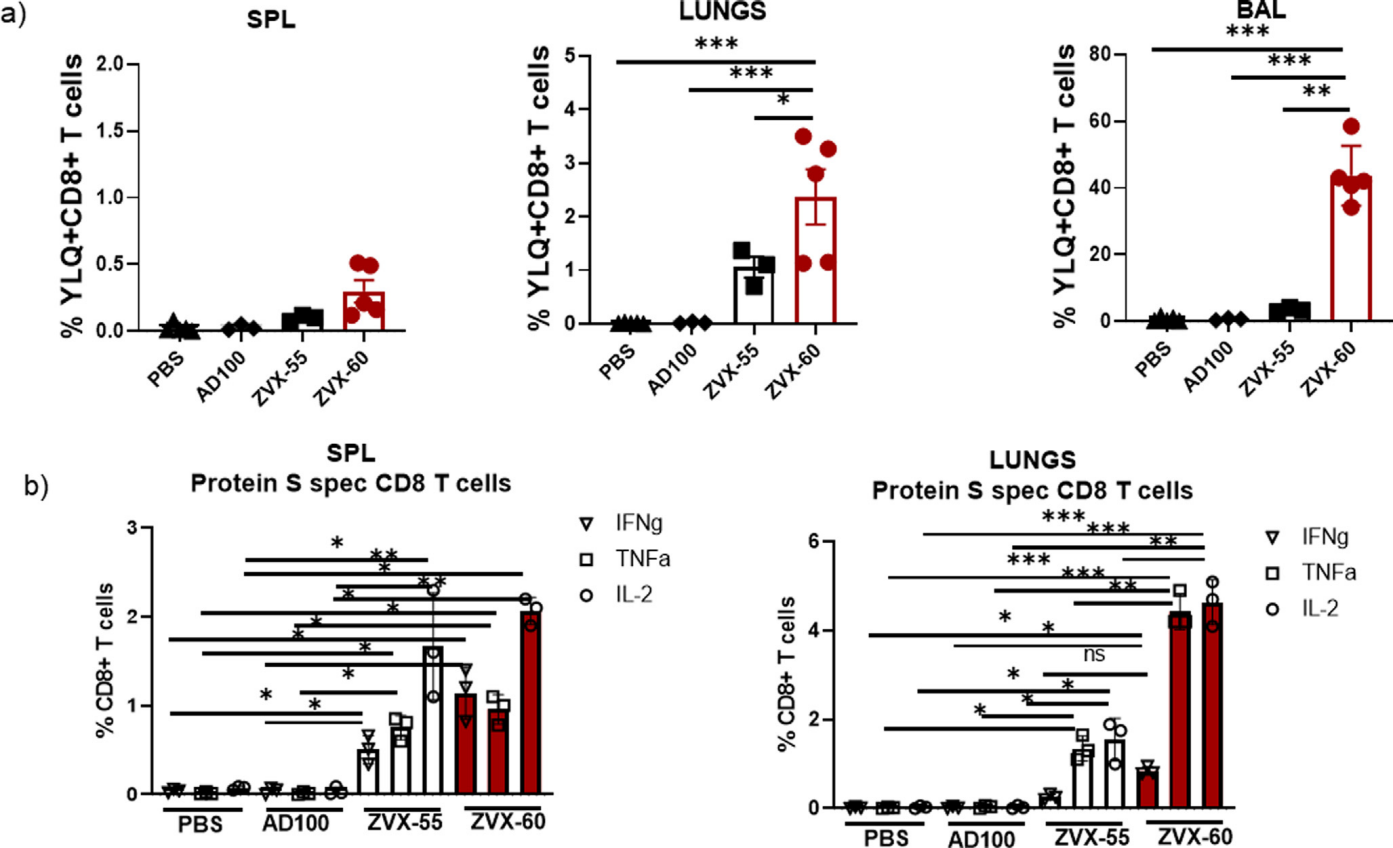
**Enhancement of S1- specific CD8 + T cell responses by Gp96-Ig-S-OX40L-Fc in the spleen, lung tissue, and BAL**. a) 5 days after the vaccination of HLA-A2 transgenic mice (n = 3–5) with the ZVX-55, ZVX-60 vaccine cells (1ug secreted gp96-Ig) or AD100 or PBS (controls), splenocytes (SPL), lung cells and bronchioalveolar lavage (BAL) cells were isolated from vaccinated and control mice (PBS). Cells were stained with HLA-A2 02–01 pentamer containing YLQPRTFLL peptides, followed by surface staining for CD45, CD3, CD4, CD8, CD69, CXCR6. Bar graphs represent percentage of the pentamer positive cells within CD8 + T cells. b) 5 days after the vaccination of C57Bl6 mice (n = 3), splenocytes and lung cells were isolated from vaccinated and control mice (PBS and AD100) and *in vitro* restimulated with S1 and S2 overlapping peptides in the presence of protein transport inhibitor, brefeldin A for the last 5 h of culture. After 20 h of culture, intracellular cytokine (IFNg, TNFa and IL-2) staining was preformed to quantify protein S-specific CD8 + T-cell responses. Cytokine expression in the presence of no peptides was considered background and it was subtracted from the responses measured from peptide pool stimulated samples for each individual mouse. Data represent at least 2 technical replicates with 3–5 independent biologic replicates per group and mean ± standard error. To compare > 2 experimental groups, 2-way analysis of variance (ANOVA) test with Holm-Sidak multiple-comparison test were applied, *p < 0.05, **p < 0.01, ***p < 0.001.

In our previous study [1] we reported that the gp96-Ig-S vaccine elicits strong SARS-CoV-2 -specific CD8 + T cell responses both systemically and locally, in the lungs, with the highest frequency of S protein-specific CD8 + T cells in the BAL of vaccinated mice. Since OX40 (CD134) and its binding partner OX40L (CD252) are key costimulatory molecules involved in the generation of protective CD8 + T-cell responses at mucosal surfaces, such as the lungs [45], we wanted to compare the effect of OX40L-Fc on gp96-Ig induced protein HLA class I-specific cross-presentation of immunodominant SARS-CoV-2 protein S epitopes. To do this, we used transgenic HLA-A 02:01 mice and HLA class I pentamers as probes to detect CD8 + T cells specific for immunodominant SARS-CoV-2 protein S epitopes: YLQPRTFLL (YLQ) (aa 269–277) in vaccinated mice (Fig. 5**a**). We found that the gp96-Ig-S-OX40L-Fc (ZVX-60) vaccine effectively induces YLQ + CD8 + T cells in the spleen, lungs, and BAL (Fig. 5**a**). As expected, co-stimulation with OX40L-Fc significantly enhanced gp96-Ig-S induced S-specific CD8 + T cell responses in lung and in the BAL (Fig. 5**a**). In addition, we evaluated ZVX-55 and ZVX-60 vaccine induced polyepitope, protein S-specific CD8 + T-cell responses. After stimulation with pooled S peptides (S1 + S2) we assess IFNγ, TNFα, and IL-2 cytokine expression in spleen and lung CD8 + T cells (Fig. 5**b**). We found that all vaccinated animals showed significantly higher magnitude of the protein S-specific CD8 + T cell responses against S1 and S2 epitopes compared to controls (Fig. 5**b**). In line with pentamer data (Fig. 5**a**), frequencies of polyepitope specific CD8 + T cells were also significantly increased in the lungs of mice vaccinated with ZVX-60 when compared to ZVX-55 vaccinated mice.

Overall, we showed that OX40L-Fc enhances not only S-specific immunoglobulin and TFH responses, but also protein S-specific CD8 + T cell responses.

## Discussion

Both humoral and cell-mediated immunity play a key role in vaccine-induced protective immunity against viral infections [46]. Therefore, any candidate COVID-19 vaccine that can induced durable and high-quality T and B-cell protective immunity is the most definitive solution to the COVID-19 pandemic. Over the last two decades, we have generated vaccines that activate innate immune responses and simultaneously induce both arms of the adaptive immune response [3]. Importantly, here we show that combining the secreted gp96-Ig vaccine with OX40L-Fc fusion protein enhances vaccine-induced cellular and antibody responses and has a high potential to be further developed for clinical use.

We and others [2], [4], [5], [6], [12], [13], [45], [46], [47] have shown that gp96-activated DC can take up antigenic proteins and, after processing, present their epitopes via both MHC I and MHC II, thereby priming antigen-specific CD8 + and CD4 + T cells, respectively. We have also confirmed that gp96-Ig serves as an antibody adjuvant when combined with recombinant proteins such as SIV gp120 [6] and induces both SIV antigen-specific T cell responses as well as anti-gp120 antibody responses. By simultaneously activating APC, NK and CD8 + cytotoxic T lymphocytes (CTL), gp96 provides a Th1 environment and is a potent adjuvant for cellular immunity generating antigen specific CTL. However, it was reported that high dose of gp96 primes a suppressive immune phenotype characterized by the preferential expansion of T regulatory cells [13], [15], [47], [48]. This is in line with our finding that 1 μg of gp96-Ig is more immunogenic than 2 μg. The activation of APC by gp96 also enhances their ability to take up antigens through endocytosis/pinocytosis and to process them for MHC II presentation to CD4 + T cells. Addition of the SIV-gp120 recombinant protein to 293-gp96-SIV-Ig-vaccination (gp96 and SIV gp120 in trans – not in a molecular complex) resulted in generation of CD4 + T helper cells for antibody production [6]. To achieve an antigen-specific cellular and humoral response using a COVID-19 gp96-Ig vaccine approach, without using recombinant S protein, we generated a cell line that expresses full length S protein in addition to secreted gp96-Ig (Fig. 1**c**) and OX40L-Fc (Fig. 1**d)**. After confirming the expression of protein S by immunofluorescence (Fig. 1**e**) and western blotting (**Suppl** Fig. 1), we concluded that S protein is expressed in the cytoplasm as well as on the cell membrane. However, as reported before, full length protein S is a highly unstable transmembrane protein with a high rate of protein cleavage occurring between the S1 and S2 domains. As a result, the soluble S1 is cleaved and S2 protein remains in the membrane. As expected, we detected S1 protein in the supernatant of vaccine cells (Fig. 1**f**). Soluble S1, in the same fashion as extracellular recombinant protein, was endocytosed by gp96-activated APC and further processed for MHC class II presentation to CD4 T cells. Detection of S protein-specific IgG antibody responses (Fig. 2**a, b**) and high frequency of activated B cells (CD19 + IgM + ) after vaccination **(**Fig. 3**a**), confirms that gp96-Ig acts as a B cell adjuvant. Since the amount of S1 in the supernatant of the ZVX-55 and ZVX-60 vaccine cells was comparable (Fig. 1**f**), we were able to test the effect of OX40L-Fc as an additional adjuvant to gp96-Ig (Fig. 2**b**). We confirmed that protein S-specific IgG responses were significantly enhanced in the combination vaccine (Fig. 2**b**). B cells are activated upon direct recognition of either soluble or membrane bound antigen (such as secreted protein S1 or membrane S2 protein). However, T cell-dependent immune responses are necessary for B cell activation, especially for generation and differentiation of germinal center (GC) B cells and production of high-affinity antibodies [22], [24]. The increase in FO B cells by gp96-Ig-S and OX40-L-Fc combination (Fig. 3) could be explained through the interaction of cognate B cells with TFH cells that promotes their accumulation at the T-B cell border. It is well established that at the time of antigen priming, upregulation of OX40 on TFH cells promotes their accumulation at the T-B border where they interact with cognate OX40-L expressing B cells [22], [49], [50]. Furthermore, OX40 signaling contributes to TFH maintenance, maturation, and migration to B follicles [22], [51]. We showed increased frequencies of TFH cells *in vivo* (Fig. 4**a**) and *in vitro* (Fig. 4**b)** after stimulation with cell-secreted gp96-Ig-S and OX40-L-Fc. We propose that OX40L-Fc in the vaccine contributes to the bidirectional OX40/OX40L signaling and promotes both TFH and B cell differentiation.

OX40 signals directly promote proliferation and survival of CD8 + T cells after antigen encounter [31], [34], [35], [52]. Also, OX40 ⁄ OX40L signaling impacts secondary expansion of memory CD8 + T cells [53], [54]*.* Most importantly, locally secreted Fc-OX40L provided superior priming of antigen-specific CD8 + T cells, compared to OX40 antibodies [36]. Vaccine-secreted Fc-OX40L increased CD127 + KLRG-1– memory precursor cells during the contraction phase, resulting in improved proliferation upon secondary antigen challenge [36]. The impressive increase in the frequency of S1-specific CD8 + T cells in the BAL after gp96-Ig-S and CD40L-Fc vaccination (Fig. 5) shows that OX40L (CD252) is involved in the generation of antigen-specific CD8 + T-cell responses at mucosal surfaces, particularly in lung tissue, which agrees with previous reports about the importance of the OX40/OX40L axis in protection against respiratory viruses [45].

All together, these data demonstrate that a cell-based vaccine co-secreting gp96-Ig-S and OX40L-Fc enhances humoral and cellular protein S-specific immune responses, as compared with gp96-Ig-S vaccine alone. These data suggest that targeting the OX40/OX40L axis has the added benefit of inducing protective immunity mediated by TFH cells, B cells and antigen-specific CD8 + T cells that potentially can recognize conserved internal components of specific pathogens, therefore conferring heterosubtypic immunity [45], [55]. This would be an ideal strategy in the context of SARS-CoV2 or any other virus that mutates its external antigens while maintaining more conserved internal antigens.

## Funding

This work was supported by Heat Biologics, Inc and by Department of Microbiology and Immunology (**NS**) and University of Miami (**NS**). DF is supported by NIH award AG023717. JK is supported by T32GM112601 (Medical Scientist Training Program).

## Declaration of Competing Interest

NS is inventor on the patent application No 62/983,783 entitled “Immune-mediated coronavirus treatments”; NS is a member of Heat Biologics COVID-19 Advisory Board. MMS is the Vice President of Research; ED is the Executive Director of Research.; PJ is the Director of Business Development, all are employed by Heat Biologics, Inc. RJ is the CEO of Pelican Therapeutics, a subsidiary of Heat Biologics, Inc. MMS, ED, PJ, RJ, and KP hold stock options in Heat Biologics, Inc. The remaining authors declare that the research was conducted in the absence of any commercial or financial relationships that could be construed as a potential conflict of interest.

## Figures and Tables

**Supplementary Fig. 4:**